# Cost of Health-Related Work Productivity Loss among Fly-In Fly-Out Mining Workers in Australia

**DOI:** 10.3390/ijerph191610056

**Published:** 2022-08-15

**Authors:** Bernard Yeboah-Asiamah Asare, Marshall Makate, Daniel Powell, Dominika Kwasnicka, Suzanne Robinson

**Affiliations:** 1 Curtin School of Population Health, Curtin University, Kent Street, Perth 6102, Australia; 2 Health Psychology, Institute of Applied Health Sciences, University of Aberdeen, Aberdeen AB25 2ZD, UK; 3 Rowett Institute, University of Aberdeen, Aberdeen AB25 2ZD, UK; 4 Faculty of Psychology, SWPS University of Social Sciences and Humanities, Aleksandra Ostrowskiego 30b, 53-238 Wroclaw, Poland; 5 NHMRC CRE in Digital Technology to Transform Chronic Disease Outcomes, Melbourne School of Population and Global Health, University of Melbourne, 333 Exhibition Street, Melbourne 3000, Australia; 6 Deakin Health Economics, Faculty of Health, Deakin University, Burwood 3125, Australia

**Keywords:** FIFO, health, absenteeism, presenteeism, productivity loss, mining

## Abstract

Sufficient knowledge on the work productivity impact of the health of fly-in fly-out (FIFO) workers in the mining sector in Australia is lacking. This study examined the impact of health and lifestyle behaviours on the work productivity of FIFO workers in the mining industry in Australia. FIFO workers completed an online questionnaire on health and work productivity loss measures. Linear regressions were used to model annual work productivity losses through absenteeism, presenteeism and total productivity loss. Workers with a high risk for health conditions were, on average, associated with 3.87% more productivity loss (absenteeism: 1.27% and presenteeism: 2.88%) than those with low risk. Workers who had multiple health risks classified as medium (3–4 health conditions) and high (5 or more health conditions) reported 1.75% and 7.46% more total productivity loss, respectively, than those with fewer multiple health risks (0–2 health conditions). Health conditions were estimated to account for an annual additional productivity cost due to absenteeism of AUD 8.82 million, presenteeism of AUD 14.08 million and a total productivity loss of AUD 20.96 million per 1000 workers. FIFO workers with high health risks experience more absenteeism, presenteeism and overall productivity loss. These measures provide strong economic justifications that could support the need for targeted workplace health interventions.

## 1. Introduction

The mining industry is a significant contributor to the Australian economy, and a significant proportion of the workforce work in fly-in fly-out (FIFO) work arrangements [1]. Under FIFO work arrangements, workers travel to work at remote places for a period of time and travel back to spend leave periods at home [2]. Workers generally work compressed day and/or night shifts and long hours of a standard 12 h [3], often separated from their families. They earn fairly higher wages than workers in other types of employment and/or industry [4]. FIFO work arrangements are also practiced in the offshore oil and gas industry around the world, notably in countries including Norway, the United Kingdom and Canada. The demands of FIFO work arrangements are indicated to contribute to a high prevalence of several health conditions and risky behaviours [5,6]. Specifically, FIFO workers report higher levels of psychological distress, poorer sleep, and more fatigue, smoke more, consume more alcohol, and are more likely to be overweight and obese than the general population [6].

Productivity losses are indicated as major economic consequences of such health problems on employers and employees [7,8], besides the associated direct medical and pharmaceutical costs/claims [8,9]. Productivity loss caused by health problems denotes output loss due to reduced labour input as a result of absenteeism (absences of a worker from work or the number of working time a worker is absent from work due to sickness) and presenteeism (present at work but limited by illness and not able to fully function) [10,11,12].

Several studies have documented physical health problems such as musculoskeletal disorders [13,14,15,16] and mental health disorders such as psychological distress, depression and anxiety disorders [13,14,15,16,17] to have high absenteeism, presenteeism and/or productivity loss costs. For instance, a study in the United States has documented that workers with poor physical health reported 1.9% more productivity loss compared to those with good physical health [18]. Among employees in Australia, psychological distress has been found to be associated with a 22% increased risk of absenteeism and an over 300% increased risk of presenteeism [13], and it is estimated to account for AUD 5.9 billion (Australian dollars) in reduced productivity in a year [19]. Fatigue and sleep-related problems have also been demonstrated to account for $15.3 billion and $21.5 billion, respectively, in productivity loss due to presenteeism per annum among workers in Japan [14]. Several studies have identified health-related behaviours, including smoking, alcohol consumption, physical activity, eating behaviours, overweight and obesity and relaxation time, as significant predictors of work-related absenteeism and/or presenteeism [9,18,20,21,22,23,24]. For instance, workers who are current smokers compared to non-smokers and physically inactive compared to physically active were found to report 2.8% and 1.9% reduced productivity, respectively, in the United States [18].

Furthermore, evidence suggests that the co-occurrence of health and risky behaviours are important contributors to productivity loss [9,18,25,26,27] and future medical claims, thereby imposing a high financial burden on employers [9]. Individuals with more health behaviours experience higher levels of absenteeism and presenteeism than those with fewer risk behaviours [26,27]. A study among workers in a large company in the United States has reported workers with five or more co-occurring health risks such as smoking, alcohol use, and physical inactivity were 12.2% less productive than workers with low (0 to 2) health risky behaviours, and the occurrence of every extra health risk accounted for a 2.4% reduction in productivity [18].

There is a limited number of studies that have evaluated the economic impact of the healthy and unhealthy behaviours of workers in the mining sector in Australia [1,28,29]. A cross-sectional study of mining workers in Australia estimated an annual cost of AUD 22 million in lost productivity in every 1000 workers attributable to seven health conditions, including stress, depression, anxiety, sleep problems, alcohol use and poor nutrition [29]. Similarly, Ling et al. established that psychological distress is associated with an annual cost of loss of work time of AUD 4.9 million in mining workers, with AUD 2.7 million due to absenteeism and AUD 2.3 million due to presenteeism [1]. Studies examining health-related productivity in the mining industry tend to focus on the entire mining workforce rather than workers on FIFO work arrangements, which are increasingly becoming the standard form of employment in the mining industry in Australia [2]. FIFO workers may differ in mental health and health-related behaviours from their counterpart mining workers who are not FIFO [30,31,32]. Current studies have largely focused on a single health condition (e.g., psychological distress or stress) [1,28] and are limited in examining the economic impact of multiple health conditions [29] and their co-occurrence among workers. Additionally, the health risk profile of workers may change over time, which will require the regular evaluation of workers’ health and accompanying economic impact. For instance, a study by Nielsen et al. found a decrease in the prevalence of psychological distress from 9% to 8% over a 6-month period among FIFO offshore oil and gas workers [33]. Furthermore, limited studies have examined the health and FIFO job characteristics that predict productivity loss.

The high prevalence of health problems and risk of unhealthy behaviours [6] reported among FIFO workers requires better workplace health and safety interventions and policies. However, it has been suggested that employers may be reluctant to uptake or support such interventions unless an economic impact on the health and safety of workers has been demonstrated [29]. Employers seek to regularly measure the financial gains workplace health interventions bring to their organizations [9] as they look to improve the health of workers and enhance work productivity. One way of demonstrating this financial benefit is to examine the impact of the health risk of workers on work productivity outcomes and/or the associated productivity cost [29,34]. Furthermore, providing a comprehensive examination of the work-related factors that promote work productivity losses may be particularly beneficial to profiling which workers are at higher risk of experiencing productivity losses and where interventions could be targeted. For this, obtaining the essential information on the health and work-related predictors of productivity outcomes is of high economic and societal significance.

This cross-sectional study aimed to examine the self-reported health and lifestyle behaviours of FIFO workers and the impact of this on work productivity in people working in FIFO work in the mining industry in Australia. Specifically, the study examined: (1) productivity losses attributable to the health and lifestyle behaviours of FIFO workers; (2) the relationship between health and lifestyle behaviours and productivity losses; (3) health and work-related predictors of productivity loss (absenteeism, presenteeism and total productivity loss); and (4) the annual cost of absenteeism, presenteeism and total productivity loss among FIFO workers.

## 2. Materials and Methods

### 2.1. Study Design and Participants

A cross-sectional study was conducted among FIFO workers in the mining industry in Australia. The resources industry in Australia employed an average of 264,700 people in 2021 [35], and around 90,000 to 11,000 have been estimated to work in FIFO roles [36]. FIFO work arrangements are predominant in Western Australia and Queensland [37], accounting for an estimated 17% of employment in the regional areas of Australia [38,39,40]. Workers travel (commonly by plane) from the cities to regional, remote areas; for instance, in Western Australia, workers travel from Perth to work in the remote areas of Pilbara, Kimberly, Goldfields-Esperance and Central Midwest regions [4]. FIFO workers in the mining sectors, including metal ore mining (such as gold, iron, lead, copper, etc.), coal mining and oil and gas) are predominately males (≈85%) and aged 25–44 years (58.6%) [39]. Workers in the mining sector commonly work on a FIFO roster of 14 days on/7 days off or 8 days on/6 days off [41].

Data were collected via an online questionnaire through the Qualtrics XM online survey software [42]. The study used a convenience (non-probability) sampling procedure to recruit a readily available FIFO sample interested in taking part in the study, which is suggested to be suitable to draw responses from a ‘mobile population’ like FIFO workers [43]. Study participants aged 18 years and above and working on FIFO arrangements in the mining industry in Australia were recruited between July and December 2021 through a large mining company in Western Australia, where promotional materials were posted at various sites and through the company’s weekly intranet communications. Study participants were also recruited through the periodic posts of promotional materials on Facebook pages of FIFO work support groups to increase diversity in the recruited study sample. The use of social media platforms in recruiting study participants has been demonstrated as an effective recruitment strategy in previous FIFO studies [40]. Study participants provided informed consent and completed the questionnaire voluntarily and anonymously. Research promotional materials invited only FIFO workers in the mining sector, and each participant acknowledged that they did work FIFO. There was no system in place to track the number of participants in the study who were identified via Facebook posts or recruited through the mining company.

### 2.2. Survey Instruments and Measures

Given the novelty of this study, there was not a previously published questionnaire that could fully answer the question of interest. In the absence of a validated questionnaire, this study drew on previously published literature that had focused on a number of relevant areas relating to health and related behaviours and productivity losses. For each of the areas, we identified relevant sources questionnaires and established scales and national guidelines in supporting the development of a specific questionnaire (available on request to the first author). The final survey consisted of 57 questions across sociodemographic and work characteristics, health and related behaviours and work productivity measures (absenteeism and presenteeism).

#### 2.2.1. Sociodemographic and Work Characteristics

Sociodemographic and work characteristics assessed included: age, sex, ethnicity, marital status, number of children, educational status, FIFO role, shift pattern, normal shift hours per day, number of consecutive days at work and at home, and the duration of working as a FIFO worker consistent with previous studies [44,45].

#### 2.2.2. Health Conditions

Health and related behaviours commonly reported among rotation workers [6] and highlighted as significant contributors to work productivity loss [29,46] were assessed using established scales and national guidelines. The health conditions included: psychological distress, physical health status, sleep condition, risky use of alcohol, physical inactivity, smoking, weight problem (low and high body mass index (BMI), and poor diet (insufficient fruit and vegetable intake).

Psychological distress was assessed using the 10-item Kessler Psychological Distress Scale-K10 [47], which assesses the negative emotional states (e.g., feeling nervous, sad, depressed, worthless, or hopeless) over the previous 30 days on a 1 to 5 Likert scale. Based on the total score of 10–50, a high risk of psychological distress was classified as a score of 22–50 [47].

Physical health status was evaluated by the 4-item physical component summary (PCS) subscale of the SF-8 Health Questionnaire [48]. Items assess the experiences of bodily pain, difficulty in doing daily work and limitation to physical activities due to physical health problems and perceived overall health status in the last 4 weeks on 5- or 6-point Likert scales. Of the potential scores of 0 to 100, a score less than 50 was indicative of poor physical health status [48].

Sleep condition was assessed using questions taken from the Pittsburgh Sleep Quality Index (PSQI); these include an item on sleep duration (“How many hours of actual sleep did you get at night during on-shift days?”) and one on sleep quality (“During the past month, during on-shift days, how would you rate your sleep quality overall?”) [49]. Poor sleep condition was classified as participants who reported a sleep duration of less than 7 h and/or rated their sleep quality as fairly to very bad [29].

Risky use of alcohol was assessed using Alcohol Use Disorders Identification Test-Concise (AUDIT-C) [42]. The 3-item (e.g., “How many standard drinks containing alcohol do you have on a typical day when drinking?”) scale using a 0 to 4 scale assessed the frequency and quantity of standard alcohol drinks intake typical for on-shift days. Of a total score of 0–12, male participants with ≥4 and female participants with ≥3 scores were deemed to engage in risky alcohol drinking behaviour [50].

On smoking status, participants were asked “Do you smoke?” and “Have you ever smoked?” and were classified as never smoked, previous smokers or current smokers.

Physical activity was assessed using the International Physical Activity Questionnaire-short form (IPAQ) [51]. IPAQ assesses the number of days and minutes per week spent engaging in mild, moderate and/or vigorous physical activities. Each activity’s weekly metabolic equivalent minutes (MET-minutes), given by the product of minutes, days and an established intensity (in METs), were computed and all added to give the total weekly physical activity [52]. Participants not achieving a minimum of 600 MET minutes per week were classified as undertaking insufficient physical activity [52].

Weight problem was evaluated by estimating the body mass index (BMI) based on participants’ self-reported weight and height. Participants recording BMI scores of <18.5 (underweight), 25–29.9 (overweight) and ≥ 30 (obese) were classified as having a weight problem.

Diet was measured based on fruit and vegetable intake. Participants were asked “How many serves of vegetables do you usually eat each day?” and “How many serves of fruit do you usually eat each day?” during on-shift days [53]. Per the Australian daily dietary guidelines on minimum daily-suggested servings, the intake of less than 2 servings of fruits and/or less than 5 servings of vegetables was classified as poor diet/nutrition [54]. Table 1 presents the full risk classifications of health and lifestyle behaviours.

#### 2.2.3. Work Productivity Loss Measures

Work productivity was assessed using the Worker Productivity and Activity Impairment-General Health (WPAI-GH) tool [55]. The WPAI-GH is a six-item validated tool that measures self-reported current employment status, work hours missed due to health problems in the last 7 days, the actual work hours in the last 7 days, and the extent of work impairment or reduced work productivity (or daily activities) due to health problems in the last 7 days [55]. This tool has been used to study productivity loss cost in the resource sector based on its reliability, shortness and capacity to estimate productivity loss cost in monetary terms [29]. Consistent with previous studies, this study adopted a measurement period of 4 weeks to limit the chance of influence of acute illnesses and workers’ rosters arrangements on self-reported study parameters [29,46]. Scoring on the items measured over a 4-week recall period were then divided by 4 to align to the 7 days of the original scoring metric [29] before computing the productivity loss measures, defined as the productivity lost at work in hours expressed in percentages and computed as per standard equations given by the WPAI-GH tool [55]. The validity and reliability of the WPAI-GH scale [55] and its use in the resources industry [29] and general population [46,56] are well demonstrated. Absenteeism was defined as a percentage of work hours missed due to health problems and calculated as:

$$\frac{\mathrm{work}\;\mathrm{hours}\;\mathrm{missed}\;\mathrm{due}\;\mathrm{to}\;\mathrm{health}\;\mathrm{problems}\;\mathrm{in}\;\mathrm{the}\;\mathrm{last}\;7\;\mathrm{days}\;}{\left(\mathrm{work}\;\mathrm{hours}\;\mathrm{missed}\;\mathrm{due}\;\mathrm{to}\;\mathrm{health}\;\mathrm{problems}+\mathrm{actual}\;\mathrm{hours}\;\mathrm{worked}\;\mathrm{in}\;\mathrm{the}\;\mathrm{last}\;7\;\mathrm{days}\right)}\;\times \;100$$

Presenteeism, defined as the percentage of impairment/reduced productivity while working due to health problems, was estimated as:

$$\frac{\mathrm{extent}\;\mathrm{of}\;\mathrm{work}\;\mathrm{impairment}\;\mathrm{or}\;\mathrm{reduced}\;\mathrm{work}\;\mathrm{productivity}\;\mathrm{in}\;\mathrm{the}\;\mathrm{last}\;7\;\mathrm{days},\;\mathrm{rated}\;0\;\mathrm{to}\;10\;}{10}\;\times \;100$$

The total productivity loss as a result of health problems measured as a combination of absenteeism and presenteeism was given as:

total prod loss (in %)=absenteeism+[(1 - absenteeism) × presenteeism]

### 2.3. Data Analysis and Cost Estimation Plan

Data were processed and analysed using STATA version 13 software (StataCorp LP, College Station, TX, USA). For descriptive purposes, categorical variables were presented in frequencies and percentages, and continuous variables in means and standard deviations. The risk of health conditions was classified into high risk and low risk for participants based on the measurement scales used [9,29] (see Table 1). Multiple health risks (having multiple health conditions) was determined for each participant and classified as low (0–2 health conditions), medium (3–4 health conditions) and high risk (5 or more health conditions) [27]. Productivity loss due to absenteeism, presenteeism and the total productivity loss was estimated for each participant and the differences in productivity loss of high health risk participants and low health risk participants were calculated as the excess work productivity loss attributable to the health conditions [29]; given as:

excess loss (%)=productivity loss in high health risk - productivity loss in low health risk

In examining the relationships between health conditions and absenteeism, presenteeism and total productivity loss, the Mann–Whitney tests were used to examine differences in the absenteeism, presenteeism and total productivity loss between the workers with high and low risks for health conditions. Furthermore, the Kruskal–Wallis test was conducted to examine differences in the absenteeism, presenteeism and total productivity loss between the workers with high, medium and low multiple health risks for health conditions.

To estimate the excess annual productivity loss cost attributable to the health conditions per worker, the percentage of excess work productivity loss was multiplied by the average earnings per year for full-time mining workers in Australia [29], given as:

$$\mathrm{cost}\;\mathrm{attributed}\;\mathrm{per}\;\mathrm{worker}=\frac{\mathrm{excess}\;\mathrm{loss}}{100}\;\times \;\mathrm{annual}\;\mathrm{salary}\;$$

The annual earning was estimated as AUD 134,323.20 based on the average weekly earnings as of May 2021 (for all workers in the mining industry taken from the Australian Bureau of Statistics) [57] multiplied by 48 working weeks per year (assumed for full-time workers: 52 weeks minus 4 weeks of annual leave) in the mining industry consistent with previous studies [1,29].

Consistent with previous studies [26,46], linear regressions, controlling for age, gender, work characteristics and co-occurrence of health risk, were also used to model excess annual work losses through absenteeism, presenteeism and total productivity loss, and annual productivity loss cost estimated per 1000 workers. Residuals were fairly normally distributed, and the plots of standardized residuals against predictor variables showed linear relationships [58,59].

*Y = α +β*_1_*age +β*_2_*gender + β*_3_*fifo roles + β*_4_*shift patterns + β*_5_*shift hours + β*_6_*consecutive days at*

*work+ β*_7_*consecutive days at home + β*_8_* years spent in FIFO + β*_9_*poor sleep condition +*

*β*_10_*smoking+ β*_11_*alcohol use + β*_12_*poor diet + β*_13_*bmi + β*_14_*insufficient physical activity +*

*β*_15_*poor public health + β*_16_*psychological distress + β*_17_*multiple health risk + u*,

where *Y* = productivity loss measure (absenteeism, presenteeism or total productivity loss).

To estimate the excess annual work productivity loss cost (due to absenteeism, presenteeism and total productivity loss) for each of the health conditions per 1000 workers, the coefficients (excess productivity loss for high risk) estimated from the regressions were multiplied by the prevalence for each health condition, the average annual salary (AUD $134,323.20) and by 1000 workers, consistent with previous studies [29,46]. Simply, the cost attributed to a health condition was given as

$$\mathrm{cost}=\frac{\mathrm{prev}\;\mathrm{of}\;\mathrm{health}\;\mathrm{condition}}{100}\;\times \;\frac{\mathrm{excess}\;\mathrm{loss}}{100}\times \;\mathrm{annual}\;\mathrm{salary}\;\times \;1000\;\mathrm{workers},\;$$

where

*prev = prevalence of a health condition,*
*excess loss = excess work productivity loss given by the regression coefficients (due to absenteeism or presenteeism or total productivity loss) attributable to an individual at high risk of a health condition,*
*annual salary = average annual salary for full-time mining; AUD $134,323.20*

#### 2.3.1. Sensitivity Analysis

The probabilistic sensitivity analyses (PSA) were done to examine the uncertainty of the study parameters and to test the robustness as well as validate the study model estimates. The PSA were done using Monte Carlo simulation to test for the uncertainty of parameter values in estimating the productivity loss costs. The estimation of the productivity loss costs was replicated with 1000 simulations, where the values for the parameters in PSA were based on the distributions and model estimates (point estimates and standard error) from the study sample data. There was no special rule for the selection of simulation trials (1000 samples) used in this study, but the selection and use of 1000 samples were made based on previous literature, which has been demonstrated to achieve convergence and accuracy for mean parameters [60,61]. The results of the PSA are presented using the scatter plot graphs and the 95% certainty intervals reported.

#### 2.3.2. Health- and Work-Related Predictors of Work Productivity Loss

The health- and work-related predictors of work productivity loss (absenteeism, presenteeism and total productivity loss) were examined using a two-part model approach [62]. This was due to the data containing a large number of zeros, with several of the study participants reporting no productivity loss during the study period. This is consistent with similar studies [26]. The first part of the model used multiple logistic regression to examine the health outcomes and work characteristics (job type, years working in FIFO arrangements, shift pattern, shift hours, consecutive days spent at home, consecutive days spent away from home) predictors of any reported productivity loss (i.e., reported productivity loss vs. no productivity loss) for the total study sample (*n* = 216). The second part of the model specified ordinary least square regressions to examine the relationships between health outcomes and work-related characteristics and productivity loss among the sample that reported positive productivity loss [62]. Three logistic regression models (one each for absenteeism, presenteeism and total productivity loss) and three ordinary least square regression models (one each for absenteeism, presenteeism and total productivity loss) were conducted, with statistical significance set at *p* < 0.05. The estimated variance inflation factor (VIF) values, to test for multi-collinearity in the models, ranged from 1.16 to 6.31.

## 3. Results

### 3.1. Background Characteristics of Study Participants

A total of 299 FIFO workers took part in the study: 83 of them did not provide sufficient data, particularly on health conditions and work productivity measures (absenteeism/presenteeism) and were excluded, leaving 216 who provided complete data to be included in the analysis. The excluded sample did not significantly differ in background characteristics from the included sample: e.g., age (mean age 39.9 ± 11.6 vs. 39.3 ± 12.2, *p* = 0.710), gender (male: 66.2% vs. 60.2%, *p* = 0.334), shift patterns (rotation shift: 56.0% vs. 56.7%, *p* = 0.920) and shift length (11.9 ± 1.7 vs. 11.8 ± 1.8 h, *p* = 0.599) (Supplementary Information S1).

The background characteristics of the study participants are shown in Table 2. The mean age of the participants was 39.9 ± 11.6 years, and the majority of the participants were males (66.2%). Most of the participants worked on a rotating shift pattern (i.e., a mix of day/night shift) (57.4%) for 12 h or more per day (86.1%) and have worked in FIFO work arrangements for 5 years or more (59.7%) (Table 2).

### 3.2. Prevalence of Risk of Health Conditions

Table 3 presents the prevalence of health conditions among study participants. All participants reported at least 1 health condition. The study participants showed a high prevalence of poor diet (96.3%), weight problems (74.5%), and poor sleep conditions (64.4%). The majority of the participants (97.7%) reported having at least 2 health conditions (Table 3).

### 3.3. Productivity Loss in Individuals with High Health Risks

The proportions of study participants reporting any missed work hours and reduced productivity due to health problems were 20.4% (*n* = 44): average work hours missed of 16.07 ± 20.34 h (range 1–96) per 4 weeks and 53.7% (*n* = 116), respectively. On average, the study participants reported 1.70% absenteeism, 3.84% presenteeism and 7.48% total productivity loss rates per week during the study period (Table 4).

Workers with a high risk of each of the health conditions reported excess (more) productivity loss compared with workers with low risk. For absenteeism, high-risk workers reported more productivity loss (on average 1.27%), ranging from 0.07% (risky alcohol use) to 2.77% (poor physical health). A Mann–Whitney test showed workers with high risk for insufficient physical activity (*z* = −2.322, *p* = 0.020), poor physical health (*z* = −2.453, *p* = 0.014) and high psychological distress (*z* = −2.959, *p* = 0.003) reported significantly higher percentage absenteeism than those with low risks (Table 5; Supplementary Information S2).

For presenteeism, high-risk workers reported more productivity loss (on average 2.88%), ranging from 0.42% (risky alcohol use) to 8.63% (poor physical health). A Mann–Whitney test showed workers with high risk for poor sleep conditions (*z* = −2.390, *p* = 0.017), smoking (*z* = −2.609, *p* = 0.009), poor physical health (*z* = −5.000, *p* < 0.001) and high psychological distress (*z* = −6.069, *p* < 0.001) reported significantly higher percentage presenteeism than workers with low risks (Table 5; Supplementary Information S2).

On total productivity loss, high-risk workers reported more productivity losses (on average 3.87%), ranging from 0.57% (risky alcohol use) to 10.71% (poor physical health). A Mann–Whitney test showed workers with high risk for poor sleep conditions (*z* = −2.220, *p* = 0.026), smoking status (*z* = −2.183, *p* = 0.029), insufficient physical activity (*z* = −2.114, *p* = 0.035), poor physical health (*z* = −4.554, *p* < 0.001) and high psychological distress (*z* = −5.432, *p* < 0.001) reported significantly higher percentage total work productivity losses than workers with low risks (Table 5; Supplementary Information S2).

**Figure 1:**
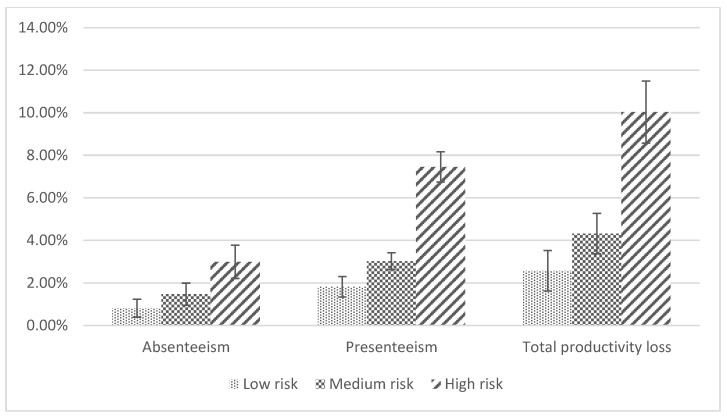
Average percentage productivity loss for each level of health risk.

The productivity loss was estimated for workers with multiple health risks, and the results showed the average percentage of absenteeism, presenteeism and total work productivity loss increased when health conditions accumulated in workers. The results are shown in Figure 1. The Kruskal–Wallis test showed there were significant differences in absenteeism (χ^2^(2) = 10.643, *p* = 0.005), presenteeism (χ^2^(2) = 25.391, *p* < 0.001) and total work productivity loss (χ^2^(2) = 23.943, *p* < 0.001) between the levels (low, medium and high) of accumulation of multiple health conditions (Supplementary Information S3). A Dunn’s test of multiple comparisons with Bonferroni adjustment showed that average percentage of productivity loss measures (absenteeism, presenteeism and total productivity loss) were significantly higher in workers with high multiple health risks (5 or more health conditions) compared to workers with low (0–2 health conditions) (*p* < 0.001) and medium (3–4 health conditions) multiple health risks (*p* < 0.001). For instance, total productivity loss increased from 2.57% in workers with low risk (0–2 health conditions) to 10.03% in workers with high risk (5 or more conditions), and compared to the low-risk workers (0–2 health conditions) (*p* < 0.001) and those with medium risk (3–4 health conditions) (*p* < 0.001), workers with high risk reported greater productivity loss of 7.46% and 5.71% respectively (Supplementary Information S3).

The cost of excess productivity loss due to absenteeism, presenteeism and total productivity loss was computed for individuals with higher levels of health risk by multiplying the percentage of excess productivity loss by the average annual wage (AUD 134, 323.20) (Table 4). The excess productivity loss due to absenteeism for the health conditions accounted for an additional average cost of AUD 1629.79 per year per worker, with the lowest of AUD 94.03 reported for risky alcohol use and the highest of AUD 3720.75 for poor physical health. The average additional cost of excess productivity loss due to presenteeism for the health conditions was AUD 3871.87 per year per worker, ranging from AUD 564.16 for risky alcohol use and AUD 11,592.09 for poor physical health. On average, excess total productivity loss (combination of absenteeism and presenteeism) for the health conditions accounted for an additional cost of AUD 5194.95 per year per worker, with the highest 3 contributors including poor physical health (AUD 14,386.01), psychological distress (AUD 8663.85), and poor diet (AUD 4741.61).

### 3.4. Productivity Loss in Individuals with High Health Risks

To estimate the independent contribution of each health conditions to absenteeism, presenteeism and overall productivity loss, linear regression was used to estimate the unstandardized coefficients (excess productivity loss) for each health risk adjusting for age, gender, work-related characteristics, and co-occurrence of health risk factors (Table 6). The excess productivity loss was multiplied by the prevalence of each health condition and multiplied by the average annual salary (AUD 134,323.20) of a full-time mining worker in Australia and by 1000 workers to estimate the productivity loss cost (due to absenteeism, presenteeism and total productivity loss) for each of the health outcomes per 1000 workers per year. All health outcomes recording excess productivity loss in high-risk workers were included in estimating each of the productivity loss costs as they showed substantial cost. The F-test also showed a significant contribution of all health outcomes to the models estimating presenteeism, F_(8, 198)_ = 10.66, *p* < 0.001 and total productivity loss, F_(8,198)_ = 6.30, *p* < 0.001, except for the model estimating absenteeism, F_(8,198)_ = 1.49, *p* = 0.164 (Table 5).

The estimated average productivity loss cost due to absenteeism was AUD 1,259,341.41 per 1000 employees per year, ranging from AUD 329,790.32 for poor physical health to AUD 4,139,303.73 for poor diet. The total annual productivity loss cost attributed to absenteeism due to seven health conditions (excluding smoking, which recorded no excess productivity loss due to absenteeism in those at high risk of smoking) was AUD 8,815,389.84 per 1000 employees.

On average, productivity loss cost due to presenteeism was AUD 1,760,040.25 per 1000 workers per year, ranging from AUD 446,812.69 for smoking to AUD 5,510,448.09 for poor diet. The total annual productivity loss cost attributed to presenteeism due to the 8 health-related risks was AUD 14,080,321.98 per 1000 workers.

The overall productivity loss cost (combination of absenteeism and presenteeism) was, on average, AUD 2,620,548.25 per 1000 workers per year, ranging from AUD 365,251.65 for smoking to AUD 8,860,697.05 for poor diet. Annually, the 8 health risks accounted for an overall productivity loss cost of AUD 20,964,385.99 per 1000 employees. The risks of poor diet, psychological distress, poor physical health, poor sleep condition and insufficient physical activity contributed significantly to employees’ excess productivity loss cost in the study sample (*p* < 0.05) (Table 6).

### 3.5. Sensitivity Analysis

**Figure 2:**
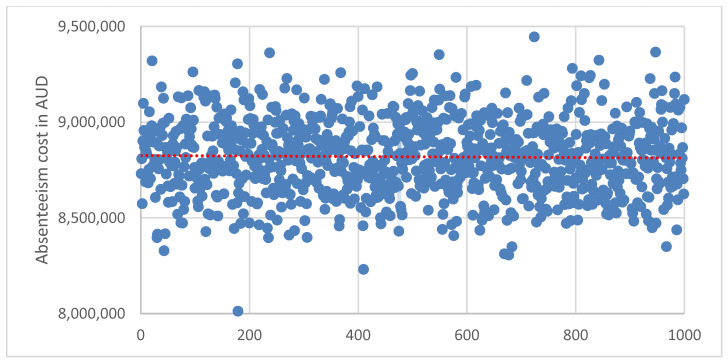
Scatter plot for probabilistic sensitivity analysis to examine the uncertainty of parameters in estimating absenteeism cost using Monte Carlo simulation to replicate the estimated cost in 1000 samples. Average absenteeism cost was AUD 8.82 (95% CI: 8.81–8.83) million.

**Figure 3:**
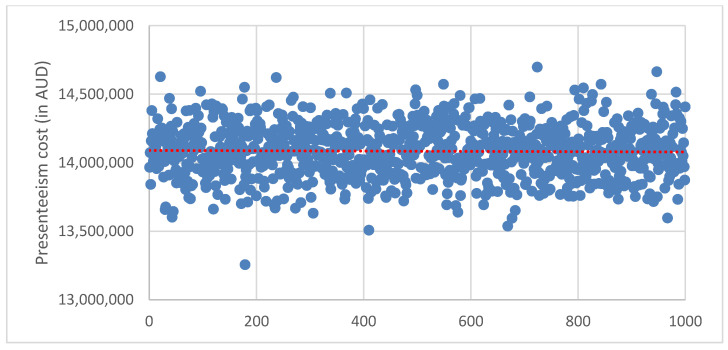
Scatter plot for probabilistic sensitivity analysis to examine the uncertainty of parameters in estimating presenteeism cost using Monte Carlo simulation to replicate the estimated cost in 1000 samples. Average presenteeism cost was AUD14.08 (95% CI: 14.10–14.07) million.

**Figure 4:**
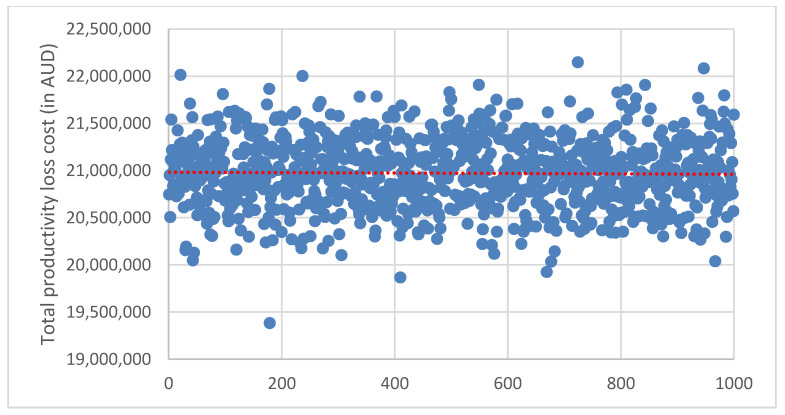
Scatter plot for probabilistic sensitivity analysis to examine the uncertainty of parameters in estimating total productivity cost using Monte Carlo simulation to replicate the estimated cost in 1000 samples. Average total productivity loss cost was AUD 20.97 (95% CI: 20.99–20.95) million.

The results from the probability sensitivity analysis to examine the uncertainty of parameters in estimating productivity loss cost using Monte Carlo simulation are presented in Figure 2, Figure 3 and Figure 4. Replicating the estimated cost in 1000 samples indicated 95% certainty intervals for the cost of absenteeism (Figure 2): AUD 8.81 million to AUD 8.83 million, presenteeism (Figure 3): AUD 14.07 million to AUD 14.10 million and total productivity loss: AUD 20.95 million to AUD 20.99 million (Figure 4).

### 3.6. Health and Work-Related Factors Associated with Productivity Loss Measures

The results of the two-part model analysis examining the health and work-related factors associated with productivity loss measures are shown in Supplementary Information S4. For absenteeism, results from the logistic regression model (−2 log-likelihood = −82.914, *p* = 0.002; pseudo R^2^ = 24.1%) showed that study participants with high risk for insufficient physical activity (OR = 2.94, 95%CI = 1.02, 8.48) and poor physical health (OR = 8.25, 95%CI = 1.88, 36.14) had higher odds of reporting any absenteeism than their counterparts with lower risk. Similarly, the odds of any absenteeism were higher among study participants who worked in production/drilling/construction/labouring roles (OR = 4.14, 95%CI = 1.09, 15.74) compared to their counterparts in management roles. Limiting the analysis to study participants who reported any absenteeism (*n* = 44), those with high risk for weight problems had low absenteeism (β = −2.48, 95% CI = −4.69, −0.26). However, the model was statistically not significant ((F_(26,17)_ = 0.90, *p* = 0.610), with adjusted R^2^ = −6.8%).

For presenteeism, logistic regression model (−2 log-likelihood = −126.347, *p* = 0.010; pseudo R^2^ = 15.3%), results showed that study participants with high risks for poor physical health (OR = 5.17, 95% CI = 1.18, 22.54) and psychological distress (OR = 4.14, 95% CI = 1.55, 11.08) had higher odds of reporting any presenteeism than their counterparts with lower risk. Limiting the analysis to study participants who reported any presenteeism (*n* = 116) in an OLS model ((F_(26,89)_ = 1.99, *p* = 0.001), with adjusted R^2^ = 18.4%), study participants with high risks for poor sleep conditions (β = 0.42, 95% CI = 0.06–0.78), poor physical health (β = 0.82, 95% CI = 0.38, 1.26) and psychological distress (β = 0.50, 95% CI = 0.12, 0.87) had high presenteeism.

For total productivity loss, logistic regression model (−2 log-likelihood = −126.048, *p* = 0.014; pseudo R^2^ = 14.9%), results showed the odds of any total productivity loss was greater among study participants with high risk for psychological distress (OR = 2.85, 95% CI = 1.07, 7.57). Limiting the analysis to study participants who reported total productivity loss (*n* = 121) in an OLS model ((F_(26,94)_ = 1.62, *p* = 0.049), with adjusted R^2^ = 11.8%),participants with insufficient physical activity (β = 0.48, 95% CI = 0.04, 0.89), poor physical health (β = 0.87, 95%CI = 0.32, 1.43) and psychological distress (β = 0.54, 95%CI = 0.08, 1.00) had high total productivity loss.

No work-related factors such as FIFO roles, shift patterns and shift hours were found to be significantly associated with presenteeism and total productivity loss (Supplementary Information S4).

## 4. Discussion

The aim of this study was to examine the impact of health and related behaviours on the work productivity of FIFO workers in the mining industry in Australia. The high prevalence of health conditions found in workers in our study reflects the extant literature, which reports high levels of poor sleep, risky alcohol use, current smoking, poor diet, high BMI, insufficient physical activity and psychological distress in FIFO workers in the resources industry [6].

The proportion of workers reporting absenteeism (20.4%) and presenteeism (53.7%) due to health problems was higher than the rates (absenteeism: 18.7% and presenteeism: 26.9%) reported in a previous study in the mining industry [29]. The differences in the measurements and the study periods could account for the observed differences in the findings. For instance, absenteeism in this current study was measured as reported hours of work missed, whereas the previous study measured absenteeism as days missed from work. However, our findings demonstrated the same trend of higher levels of presenteeism than absenteeism reported in the mining industry [1,29] and in the general working population, e.g., [26,46]. Presenteeism in the mining sector has been associated with the mining work culture and lifestyle, long working hours and fatigue [7].

Our study found excess productivity loss due to absenteeism, presenteeism and total productivity loss for health and related behaviours were highest for those reporting poor physical health and psychological distress. Not surprisingly, workers experiencing poor physical health reported excess absenteeism (2.77%) and presenteeism (8.63%) and were 10.71% less productive than workers reporting better physical health. A previous study found mining workers reporting poor physical health conditions such as musculoskeletal disorders (back, neck or spine injuries) to be 7.12% less productive than those who did not report such conditions [29]. In our study, workers experiencing a high risk of psychological distress also reported high absenteeism (2.07%) and presenteeism (4.75%) and were 6.45% less productive than workers experiencing low risk. Similar findings were reported in a previous study, demonstrating that mining workers who experience a high risk of mental health conditions were significantly less productive than workers who did not experience such conditions [29]. Another study reported psychological distress contributed to high levels of absenteeism and presenteeism among mining workers in Australia [1]. Consistent with previous studies [18,26], we reported that health risk factors including poor sleep, smoking, and insufficient physical activity were significantly associated with lower productivity in high-risk workers than in low-risk workers.

Our study found productivity loss increased with an increased number of health risks per worker. Workers with medium risk (3–4 health conditions) and high risk (5 or more conditions) compared to the low-risk workers reported lower productivity. Similar findings were also reported in the general working population in Australia [27] and the United States [18,20,26].

The number and type of health risks that were independently associated with absenteeism, presenteeism and total productivity loss differed after controlling for other covariates. A high risk of psychological distress was found to be associated with presenteeism and total productivity loss but not associated with absenteeism. Consistent with our findings, other studies reported that psychological distress was significantly associated with presenteeism and total productivity loss in the general working population [13,46,63]. The negative impact of mental health disorders on work productivity has been well documented in the general working population [9,16,21,64,65]. The severity of mental health symptoms such as impaired concentration, decision-making, communication, and social/mental interactions are indicated to drive productivity loss [63,66,67], and as such, the successful treatment of symptoms and mental health promotion interventions could substantially reduce productivity losses [65]. It has been noted that workers experiencing high distress tend to be associated with higher presenteeism than absenteeism [68,69]. Again, stigma and fear of job loss surrounding mental health disorders are suggested to prevent workers from making known and taking sickness absences due to their mental health status, thereby experiencing more presenteeism [69]. High levels of psychological distress are reported in FIFO workers [6], and seeking help for mental health is suggested to be low in the mining sector, citing fears of bullying, stigmatisation and job losses [39], worsening the mental health problems. This could account for high levels of productivity losses. The findings of our study and the broader published literature [6] suggest the need for employers to promote mental health and wellbeing and to reduce the high levels of psychological distress, including by taking measures that promote/support mental health help-seeking behaviour among FIFO workers. Ebert and Strehlow suggest that active on-site counselling and support could reduce psychological distress [70].

Consistent with the findings of previous studies among the general working populations [68,71,72], our study found poor physical health to be independently associated with absenteeism, presenteeism and total productivity loss. Poor physical health has been established as a significant contributor to high levels of absenteeism and presenteeism [18,72]. Poor physical health is indicated to limit work, particularly for those whose tasks demand strength and manual skill [18], as is the case with some mining jobs. Poor physical health conditions may also require off-the-job time to seek regular medical care and treatment [26].

Our study, similar to the published literature [73,74], demonstrates that poor sleep impacts on presenteeism, and it is associated with impaired concentration/attention, memory [74,75], fatigue [75], and worsened social/interpersonal interactions [74,76]. With FIFO workers experiencing sleep problems and fatigue, particularly during on-shift days [6], our findings suggest the need for employers to provide suitable environments that promote better sleep to enhance work productivity. However, poor sleep was found not to be associated with absenteeism and total work productivity loss, as has been documented in other studies [59,68]. Our study had a relatively small sample of participants reporting absenteeism, which could give rise to statistically insignificant relationships. Furthermore, it has been suggested that poor sleep is often associated with underlying poor physical health, which contributes to such individuals (poor sleepers) taking more sickness absence [68,77]. As in the extant literature [6], a high proportion of the participants reported good physical health in our study, which could explain the insignificant association of poor sleep with absenteeism.

In line with our findings, insufficient physical activity has also been reported to be associated with absenteeism and total productivity loss among the general working population [26,46]. Physical inactivity is indicated to be a risk factor for poor physical health conditions, several of which contribute to absenteeism due to sickness [78] and the need to take time off to seek medical treatment and recover. Sufficient physical activity [79] is indicated to mitigate mental health and stress, which could help reduce work productivity losses [80]. Our findings point toward the need for suitable health and lifestyle workplace interventions to encourage and sustain physical activity among workers, which may reduce work productivity loss [81,82].

Our study found workers in production/construction/drilling/labouring roles were associated with higher odds of absenteeism. However, no work-related factors were significantly associated with presenteeism and total productivity loss. Supporting our study in part, a previous study in Australia has found mining job roles such as technicians, tradespeople, machinery drivers and operators, and duration spent working in mining to be significantly associated with both absenteeism and presenteeism due to psychological distress [1]. The disparity in sample sizes and measurement tools between our study and the previous study could account for the observed differences. For instance, the previous study with a larger sample measured absenteeism and presenteeism in days using single items. Working in manual roles such as production/construction/drilling/labouring may come with high physical job demands, which are associated with sickness and absence from work [72]. It has been noted that workers engaged in ‘physically demanding’ jobs tended to report more absenteeism than presenteeism as they may have fewer chances to adjust their tasks to their health status compared to those engaged in ‘mentally demanding’ jobs, who may have more chances to momentarily adjust their job or speed [72]. The significant contributions of job-related characteristics to work productivity loss have been well documented [72,83]. Additional studies may be required to further explore the work-related factors that significantly contribute to less productivity in FIFO workers.

In our study, seven of the health and related behaviours (excluding smoking) were estimated to account for an excess of AUD 8.82 million in productivity loss due to absenteeism per 1000 employees per year, whereas all 8 health and related behaviours accounted for an excess of AUD 14.08 million in productivity loss due to presenteeism per 1000 employees per year. Overall, the 8 health and related behaviours were estimated to account for an excess productivity cost of approximately AUD 20.96 million per 1000 employees per year. A previous study has also estimated an additional cost of AUD 22.1 million from 7 health risk factors, including sleep condition, short-term alcohol use, poor nutrition, anxiety and depression and AUD 7.9 million from chronic health conditions including migraine and back, neck and spinal problems per 1000 mining employees per year [29]. The probabilistic sensitivity analysis to examine the uncertainty of model parameters returned certainty intervals for the estimated productivity loss costs.

The findings of our study have provided a strong indication of the economic implications of high health risk in FIFO workers. Workplace interventions could improve health and wellbeing among workers and may reduce work productivity loss [81,82]. Our findings have highlighted the financial basis for the significance of and decisions/justifications for workplace health interventions. The significant associations identified between a number of health risk and productivity loss measures provide the basis for targeted workplace health interventions and the basis for the evaluation of the impact of such interventions.

### Limitations

Some limitations to the study are acknowledged. The use of cross-sectional design limits the causal interpretation of the study findings. Secondly, the study relied on self-reported data (health conditions, absenteeism and presenteeism) over a recall period of 4 weeks. As such, there could be issues with recall bias and the under- and/or overestimation of study parameters. Further, the estimation of productivity loss cost was based on the estimated average weekly wage for a full-time worker in the mining industry; however, wages may differ between job roles (e.g., management vs. machinery operator/driver) and employment type (e.g., full time vs. part-time). This approach is consistent with previous studies in the resources industry [29] and general employment settings [9,46]; however, obtaining original data on the health conditions, time (days/hours) missed from work and wages from organizations may present close to realistic estimates. Limitations of the sampling technique used in this study are acknowledged. First, the non-probability or convenient sampling of the study participants may have the potential of sampling bias affecting the representativeness of the study sample. Secondly, the study recruited participants from two main sources, a mining company and posts on Facebook, which have the potential of sampling bias, particularly if sampling is mainly from the mining company, limiting the generalizability of the study findings. There was no system in place to track the number of participants in the study who were identified via Facebook posts or the mining company to observe any possible systematic differences in data sources. Again, recruiting through posts on Facebook have the potential to include non-FIFO workers in the study sample, though the use of social media platform in FIFO studies has been demonstrated [40]. The study also included a convenient small sample size. However, our study sample was reflective of the profile of the FIFO work population in Australia as mostly males, middle-aged and on a roster of 8 days at work, similar to that reported in a large sample study [45]. We found no significant work-related characteristics associated with presenteeism and total productivity loss, and using a larger sample size may suggest otherwise [1]. The study was conducted among FIFO workers in the mining sector whose work arrangements are unique with long shift patterns and leave periods between work periods and reported higher levels of health-related risks than the general population [6]; as such, generalising the results of this study to other work settings may be limited.

## 5. Conclusions

The study contributes to evidence on how to measure the productivity loss cost of health outcomes to inform and justify the need for and evaluate workplace health interventions. The study provides information on the impact of multiple health risk factors on absenteeism, presenteeism and overall work productivity loss among FIFO workers in the mining industry. We have also provided information on health-related productivity loss, adjusting for relevant work-related characteristics, which may significantly impact health and productivity. Our study also contributes to the growing evidence of the substantial contribution of presenteeism to productivity loss.

The study found that levels of absenteeism and presenteeism in an assessed sample of FIFO workers were high. The study also suggested that FIFO workers with high risk and multiple health conditions experienced higher absenteeism, presenteeism and overall productivity loss than those at lower risks and with fewer conditions. Overall, FIFO workers with high health risks (of poor sleep, poor diet, smoking, risky alcohol use, weight problems, insufficient physical activity, poor physical health and high psychological distress) were estimated to account for a total of AUD 20.96 million per 1000 workers per year in additional productivity cost. High risks for insufficient physical activity, poor physical health and working in production/drilling/construction/laboring roles were significantly associated with absenteeism, whereas high risks for poor sleep, poor physical health and psychological distress were significantly associated with presenteeism. Overall productivity loss was associated with insufficient physical activity, poor physical health and psychological distress. There is a strong economic basis that could support the need for targeted/prioritised workplace health interventions and the basis for the evaluation of the impact of those interventions. Further studies exploring workplace health interventions could include prior and regular analysis of productivity loss cost to inform the effectiveness of such interventions at improving the health and wellbeing of FIFO workers and reducing work productivity and cost.

## Figures and Tables

**Table 1:** High- and low risk classification for health conditions.

Health Condition	High-Risk Criteria	Low-Risk Criteria
Psychological distress	K10 scores of 22–29 (high) and 30–50 (very high) levels	K10 scores of 10–15 (low) and 16–21 (medium) levels
Poor physical health	Scores of less than 50 on the PCS of SF-8 Health scale	Scores of less than 50 on the PCS of SF-8 Health scale
Poor sleep condition	Sleep duration less than 7 h and/or poor sleep quality	Sleep duration of 7 or more hours and/or better sleep quality
Risky alcohol use	AUDIT-C score of ≥4 among men and ≥3 among women	AUDIT-C score of <4 among men and <3 among women
Smoking	Currently smoking	Non-or ex-smokers
Insufficient physical activity	Metabolic equivalent minutes (MET minutes) of less than 600 per week	Metabolic equivalent minutes (MET minutes) of ≥ 600 per week
Weight problem	BMI < 18.5(underweight), BMI = 25–29.9 (overweight) and BMI ≥ 30 (obese)	BMI = 18.5–24.9
Poor diet/nutrition	Intake of less than 2 servings of fruits and/or less than 5 servings of vegetables	Intake of more than 2 servings of fruits and/or 5 servings of vegetables

**Table 2:** Distribution of demographics and work-related characteristics of FIFO workers (*N* = 216).

Personal Characteristics	Frequency (*n*)	Percent (%)
Age in year		
≤34	82	38.0
35–44	67	31.0
≥45	67	31.0
Gender		
Male	143	66.2
Female	73	33.8
Ethnicity		
Caucasian/white	183	84.7
Other	33	15.3
Relationship status		
Single/never married	43	19.9
Married	93	43.1
Separated/divorced/widowed	25	11.6
De-facto/co-habiting/civil partnership	52	23.0
Other	3	1.4
Educational status		
Primary/secondary education and equivalent	70	32.4
Trade/apprentice	45	20.8
TAFE/college	60	27.8
Bachelor’s degree	30	13.9
Postgraduate degree	11	5.1
FIFO role		
Management/administration/services	54	25.0
Professional	27	12.5
Maintenance/technician	39	18.1
Production/drilling/construction/labourer	45	20.8
Machinery operator and driver	35	16.2
Catering	10	4.6
Other	6	2.8
Shift patterns		
Rotation shift (mixture of day/night shift)	124	57.4
Regular shift (fixed day/night)	92	42.6
Shift length		
<12 h	30	13.9
≥12 h	186	86.1
Consecutive days spent at work		
<8 days	43	19.9
8–14 days	156	72.2
15+ days	17	7.9
Consecutive days spent at home		
<8 days	187	86.6
8–14 days	29	13.4
FIFO duration		
<5 yrs	87	40.3
5–9 yrs	46	21.3
10+ yrs	83	38.4

**Table 3:** Prevalence of risk of health conditions.

Health Condition	High-Risk Frequency (*n*)	Percent (%)
Poor sleep condition	139	64.4
Risky alcohol use	74	34.3
Currently smoking	57	26.4
Poor diet	208	96.3
Weight problem	161	74.5
Insufficient physical activity	58	26.9
Poor physical health	19	8.8
Psychological distress	72	33.3
How many health conditions reported		
1	5	2.3
2	39	18.1
3	67	31.0
4	53	24.5
5 or more	52	24.1

**Table 4:** Work productivity loss measures in study participants.

Measures	Frequency (*n*), Mean ± SD	Percent (%)
Absenteeism		
Yes	44	20.4
No	172	79.6
Work hours missed per 4 weeks	16.07 ± 20.34 h (range 1–96)	
Average absenteeism rate (per week)	1.70 ± 5.36% (range 0–33.3)	
Presenteeism		
Yes	116	53.7
No	100	46.3
Reduced work productivity (ranked 0–10) per 4 weeks		
0	100	46.3
1–2	64	29.6
3–4	32	14.8
≥5	20	9.3
Average presenteeism rate (per week)	3.84 ± 5.33% (range 0–22.5)	
Average total productivity loss rate (per week)	7.48 ± 10.20% (range 0–40)	

**Table 5:** Average percentages of absenteeism, presenteeism and total productivity loss and annual excess cost attributed to health risks per worker (*N* = 216).

	Percent Absenteeism Due to Health				Percent Presenteeism Due to Health				Percent Total Productivity Loss			
Health Conditions	High Risk	Low Risk	Excess	Cost Per Year	High Risk	Low Risk	Excess	Cost per year	High Risk	Low Risk	Excess	Cost Per Year
Poor sleep condition	2.07	1.04	1.03	1383.53	4.64	2.40	2.24 **	3008.84	6.43	3.36	3.07 *	4123.72
Risky alcohol use	1.75	1.68	0.07	94.03	4.12	3.70	0.42	564.16	5.71	5.14	0.57	765.64
Current smoking	1.99	1.60	0.39	523.86	5.70	3.18	2.52 **	3384.94	7.37	4.61	2.77 *	3720.75
Poor diet	1.77	0.07	1.70	2283.49	3.92	1.88	2.04	2740.19	5.47	1.94	3.53	4741.61
Weight problems	1.77	1.51	0.26	349.24	4.02	3.32	0.70	940.26	5.56	4.69	0.86	1155.18
Insufficient physical activity	2.73	1.32	1.41 *	1893.96	5.13	3.37	1.76	2364.09	7.52	4.54	2.98 *	4002.83
Poor physical health	4.23	1.46	2.77 *	3720.75	11.71	3.08	8.63 ***	11,592.09	15.11	4.40	10.71 ***	14,386.01
Psychological distress	3.08	1.01	2.07 **	2789.49	7.01	2.26	4.75 ***	6380.35	9.64	3.19	6.45 ***	8663.85

* p < 0.05; ** *p* < 0.01; *** *p* < 0.001 from Mann–Whitney test. Australian dollar (AUD) 134,323.20 based on the average weekly earnings per worker as of May 2021.

**Table 6:** Estimates of loss in productivity per year by health indicators per 1000 FIFO workers (N = 216).

Health Conditions	Prevalence of High Risk (%)	Excess Absenteeism (%)	Lost Productivity Cost Per 1000 (AUD)	Excess Presenteeism (%)	Lost Productivity Cost Per 1000 (AUD)	Excess Total Productivity Loss (%)	Lost Productivity Cost per 1000 (AUD)
Poor sleep condition	64.4	1.41	1,219,708.39	2.17 *	1,877,139.86	3.28 *	2,837,335.82
Risky alcohol use	34.3	0.93	428,477.58	1.48	681,878.29	2.26	1,041,246.58
Smoking	26.4	−0.07	-	1.26	446,812.69	1.03	365,251.65
Poor diet	96.3	3.20	4,139,303.73	4.26 *	5,510,448.09	6.85 *	8,860,697.05
Weight problems	74.5	1.00	1,000,707.84	1.50	1,501,061.76	2.21	2,211,564.33
Insufficient physical activity	26.9	1.64	592,580.23	2.54 **	917,776.70	3.88 **	1,401,958.10
Poor physical health	8.8	2.79	329,790.32	9.05 ***	1,069,749.96	11.10 ***	1,312,069.02
Psychological distress	33.3	2.47 *	1,104,821.75	4.64 ***	2,075,454.63	6.56 ***	2,934,263.44

* *p* < 0.05; ** *p* < 0.01; *** *p* < 0.001. Adjusted for age, sex, job type, years in FIFO, shift pattern, shift hours, consecutive days spent at home, consecutive days spent at home, and co-occurrence of multiple health risks.

## Data Availability

The data that support the findings of this study are not publicly available due to ethical and privacy restrictions but are available upon request from the corresponding author on reasonable request.

## Supplementary Materials

The following supporting information can be downloaded at: https://www.mdpi.com/article/10.3390/ijerph191610056/s1, Supplementary Information S1: Differences between included and excluded study samples; Supplementary Information S2: Mann–Whitney tests were used to examine differences in productivity loss measures between the workers with high and low risk for health conditions; Supplementary Information S3: Kruskal–Wallis test of differences in work productivity loss measures between multiple health risk groups; Supplementary Information S4: Health and work-related predictors of absenteeism, presenteeism and total productivity loss.
