# Supplementary material for: Cost of Health-Related Work Productivity Loss among Fly-In Fly-Out Mining Workers in Australia
Source: Int J Environ Res Public Health. 2022 Aug 15;19(16):10056. doi: 10.3390/ijerph191610056 (PMC9408090; doi:10.3390/ijerph191610056)
Supplement: Supplementary file 1 [file ijerph-19-10056-s001.zip › Supplementary Information S1.pdf]

Supplementary Information S1

Table S1. Differences between included and excluded study samples

Characteristics	Included sample (n=216)	Excluded sample (n=83)	p-value
Age (in years)	39.9±11.6	39.3±12.2	0.710
Sex			0.334
Male	143(66.2)	50(60.2)	
female	73(33.8)	33(39.8)	
Ethnicity			0.658
Caucasian/White	183(84.7)	72(86.8)	
Other	33(15.3)	11(13.2)	
Marital status			0.999
Single/never married	43(19.9)	16(19.3)	
Married	93(43.1)	36(43.4)	
Divorced/separated/widowed	25(11.6)	10(12.1)	
De-facto/co-habiting/civil partnership/other	55(25.5)	21(25.3)	
Educational status			0.850
Secondary education/A-Levels/O GCSE/O-level or equivalent	67(31.0)	21(31.3)	
Trade/Apprentice	45(20.8)	17(25.4)	
TAFE/College/Diploma	60(27.8)	16(23.9)	
University degree/Other	44(20.4)	13(19.4)	
FIFO role			0.694
Management	33(15.3)	8(11.9)	
Professional	27(12.5)	12(17.9)	
Maintenance/Technician	39(18.1)	13(19.4)	
Catering	10(4.6)	6(9.0)	
Production/Drilling/construction	36(16.7)	9(13.4)	
Machinery operator and driver	35(16.2)	9(13.4)	
Labourer	9(4.2)	4(6.0)	
Administration/services/Other	27(12.5)	6(9.0)	
Shift pattern (n=283)			0.920
Rotation shift (mixture of day and night shift)	121(56.0)	38(56.7)	
Regular shift/Other	95(44.0)	29(43.3)	
Shift length			0.688
<12 hrs	30(13.9)	12(18.2)	
12	129(59.7)	37(56.1)	
>12	57(26.4)	17(25.8)	
