# Supplementary material for: Cost of Health-Related Work Productivity Loss among Fly-In Fly-Out Mining Workers in Australia
Source: Int J Environ Res Public Health. 2022 Aug 15;19(16):10056. doi: 10.3390/ijerph191610056 (PMC9408090; doi:10.3390/ijerph191610056)
Supplement: Supplementary file 1 [file ijerph-19-10056-s001.zip › Supplementary Information S2.pdf]

Supplementary Information S2

Table S2a. Mann-Whitney analysis of the differences in absenteeism for the risk of health conditions

Health conditions	Percent absenteeism due to health				
	**High risk**	**Low risk**	**Excess**	**z**	**p-value**
Poor sleep condition	2.07	1.04	1.03	-1.555	0.120
Risky alcohol use	1.75	1.68	0.07	0.015	0.988
Current Smoking	1.99	1.60	0.39	-1.510	0.131
Poor diet	1.77	0.07	1.70	-0.709	0.479
Weight problems	1.77	1.51	0.26	-0.348	0.728
Insufficient physical activity	2.73	1.32	1.41	-2.322	0.020
Poor physical health	4.23	1.46	2.77	-2.453	0.014
Psychological distress	3.08	1.01	2.07	-2.959	0.003

Table S2b. Mann-Whitney analysis of the differences in presenteeism for the risk of health conditions

Health conditions	Percent presenteeism due to health				
	**High risk**	**Low risk**	**Excess**	*z*	**p-value**
Poor sleep condition	4.64	2.40	2.24	-2.390	0.017
Risky alcohol use	4.12	3.70	0.42	-0.524	0.601
Current Smoking	5.70	3.18	2.52	-2.609	0.009
Poor diet	3.92	1.88	2.04	-1.011	0.312
Weight problems	4.02	3.32	0.70	-0.528	0.597
Insufficient physical activity	5.13	3.37	1.76	-1.910	0.056
Poor physical health	11.71	3.08	8.63	-5.000	<0.001
Psychological distress	7.01	2.26	4.75	-6.069	<0.001

Table S2c. Mann-Whitney analysis of the differences in total productivity loss for the risk of health conditions

Health conditions	Percent total productivity loss due to health				
	**High risk**	**Low risk**	**Excess**	*z*	**p-value**
Poor sleep condition	6.43	3.36	3.07	-2.220	0.026
Risky alcohol use	5.71	5.14	0.57	-0.877	0.380
Current Smoking	7.37	4.61	2.77	-2.183	0.029
Poor diet	5.47	1.94	3.53	-1.164	0.244
Weight problems	5.56	4.69	0.86	-0.421	0.674
Insufficient physical activity	7.52	4.54	2.98	-2.114	0.035
Poor physical health	15.11	4.40	10.71	-4.554	<0.001
Psychological distress	9.64	3.19	6.45	-5.432	<0.001
