# Supplementary material for: Cost of Health-Related Work Productivity Loss among Fly-In Fly-Out Mining Workers in Australia
Source: Int J Environ Res Public Health. 2022 Aug 15;19(16):10056. doi: 10.3390/ijerph191610056 (PMC9408090; doi:10.3390/ijerph191610056)
Supplement: Supplementary file 1 [file ijerph-19-10056-s001.zip › Supplementary Information S3.pdf]

Supplementary Information S3

Table S3a: Kruskal-Wallis test of differences in work productivity loss measures between multiple health risk groups

	Multiple health risk	Productivity loss (%)	X^2(df)	*p*-value
Absenteeism			10.643(2)	0.005*
	Low	0.81		
	Medium	1.47		
	High	2.99		
Presenteeism			25.391(2)	<0.001*
	Low	1.82		
	Medium	3.02		
	High	7.45		
Total productivity loss			23.943(2)	<0.001*
	Low	2.57		
	Medium	4.32		
	High	10.03		

*significant at p<0.05

Table S3b: Dunn's test with bonferroni adjustment nonparametric pairwise multiple comparison of work productivity loss measures between multiple health risk groups

	Multiple health risk	Excess productivity loss (%)	*p*-value
Absenteeism			
	Medium vs low	0.66	0.711
	High vs low	2.18	0.005*
	High vs medium	1.52	0.007*
Presenteeism			
	Medium vs low	1.20	0.120
	High vs low	5.63	<0.001*
	High vs medium	4.43	<0.001*
Total productivity loss			
	Medium vs low	1.75	0.137
	High vs low	7.46	<0.001*
	High vs medium	5.71	<0.001*

*significant at p<0.05
