# Supplementary material for: Cost of Health-Related Work Productivity Loss among Fly-In Fly-Out Mining Workers in Australia
Source: Int J Environ Res Public Health. 2022 Aug 15;19(16):10056. doi: 10.3390/ijerph191610056 (PMC9408090; doi:10.3390/ijerph191610056)
Supplement: Supplementary file 1 [file ijerph-19-10056-s001.zip › Supplementary Information S4.pdf]

Supplementary Information S4

Table S4a. Health and work-related predictors of any absenteeism (based on logistic regression using the total study sample) and percent absenteeism (based on least squares regression limited to study sample with positive percent absenteeism)

**Parameters**	**Logistic regression of any absenteeism** (N=216)		**Least square regression of percent absenteeism (n=44)**	
	**OR(95%CI)**	**p-value**	**β(95%CI)**	**p-value**
Age in years				
<35	1		Ref	
35-44	0.58 (0.21, 1.61)	0.293	-0.41(-2.03, 1.21)	0.619
45-54	0.28(0.07, 1.20)	0.087	-0.88(-3.06, 1.30)	0.430
55+	0.09(0.01, 0.73)	0.024	1.14(-2.25, 4.53)	0.510
Sex				
Male	1		Ref	
Female	1.86(0.75, 4.65)	0.183	-0.98(-2.31, 0.35)	0.149
FIFO role				
Management	1		Ref	
Professional	1.05(0.21, 5.21)	0.951	2.09(-0.22, 4.40)	0.076
Maintenance/Technician	2.62(0.59, 11.54)	0.204	0.57(-1.78, 2.91)	0.637
Production/Drilling/Construction/Labourer	4.14(1.09, 15.74)	0.037	1.53(-0.63, 3.68)	0.165
Machinery operator and driver	4.29(1.00, 18.37)	0.050	1.21(-1.03, 3.44)	0.290
Catering/Other	1.59(0.20, 12.69)	0.661	2.31(-0.72, 5.34)	0.135
FIFO duration in years				
5	1		Ref	
5-9	0.79(0.28, 2.25)	0.661	0.03(-1.38, 1.45)	0.965
10+	0.37(0.12, 1.15)	0.085	1.88(-0.03, 3.79)	0.053
Shift pattern				
Regular shift	1		Ref	
Rotation shift/other	1.15(0.47, 2.82)	0.763	0.62(-0.78, 2.02)	0.388
Shift hours				
12 hrs	1		Ref	
12 hrs and more	1.78(0.43, 7.36)	0.424	1.11(-1.10, 3.33)	0.325
Consecutive days spent at work				

8	1		Ref	
8-14 days	0.67(0.23, 1.91)	0.452	-1.05(-2.48, 0.38)	0.149
15+ days	0.27(0.03, 2.76)	0.272	1.81(-1.49, 5.11)	0.283
Consecutive days spent at home				
8 days	1		Ref	
8-14 days	0.69(0.15, 3.26)	0.638	-1.29(-3.90, 1.31)	0.330
Poor sleep condition	1.68(0.52, 5.46)	0.387	-0.47(-2.08, 1.15)	0.571
Risky alcohol use	0.95(0.32, 2.80)	0.922	0.05(-1.48, 1.58)	0.1949
Smoking	0.71(0.21, 2.42)	0.588	-1.28(-3.02, 0.46)	0.149
Poor diet	3.80(0.31, 46.94)	0.298	2.22(-1.95, 6.40)	0.297
Weight problems	1.50(0.46, 4.93)	0.500	-2.48(-4.69, -0.26)	0.028
Insufficient physical activity	2.94(1.02, 8.48)	0.046	-0.57(-2.41, 1.26)	0.541
Poor physical health	8.25(1.88, 36.14)	0.005	0.49(-1.29, 2.27)	0.591
Psychological distress	1.76(0.55, 5.60)	0.340	-0.20(-1.69, 1.29)	0.790
-2 Log likelihood or regression *F* statistic	-82.914		F(26, 17)=0.90	
P value for -2 log likelihood or regression *P* value	0.002		0.610	
Pseudo R^2 or adjusted R^2	0.241		-0.068	

Adjusted for age, sex and co-occurrence of multiple health risk

Table S4b. Health and work-related predictors of any presenteeism (based on logistic regression using the total study sample) and percent presenteeism (based on least squares regression limited to study sample with positive percent presenteeism)

Parameters	Logistic regression of any presenteeism (N=216)		Least square regression of percent presenteeism (n=116)	
	**OR(95%CI)**	**p-value**	**β(95%CI)**	**p-value**
Age in years				
<35	1		Ref	
35-44	0.81(0.36, 1.83)	0.610	-0.14(-0.49, 0.22)	0.451
45-54	1.03(0.36, 2.93)	0.959	-0.13(-0.55, 0.28)	0.532
55+	0.51(0.16, 1.58)	0.243	-0.02(-0.55, 0.51)	0.939
Sex				
Male	1		Ref	
Female	0.99(0.47, 2.11)	0.985	-0.01(-0.33, 0.31)	0.955
FIFO role				
Management	1		Ref	
Professional	1.81(0.62, 5.29)	0.280	0.17(-0.30, 0.65)	0.471
Maintenance/Technician	2.21(0.79, 6.19)	0.130	0.07(-0.37, 0.52)	0.745
Production/Drilling/Construction/Labourer	1.49(0.59, 3.78)	0.404	0.07(-0.34, 0.49)	0.725
Machinery operator and driver	1.74(0.60, 5.05)	0.312	0.21(-0.25, 0.68)	0.365
Catering/Other	1.69(0.44, 6.55)	0.447	0.13(-0.48, 0.73)	0.677
FIFO duration in years				
5	1		Ref	
5-9	0.87(0.37, 2.02)	0.744	-0.00(-0.35, 0.35)	0.996
10+	0.50(0.22, 1.18)	0.113	-0.13(-0.49, 0.23)	0.485
Shift pattern				
Regular shift	1		Ref	
Rotation shift/other	1.55(0.77, 3.12)	0.223	0.13(-0.15, 0.41)	0.357
Shift hours				
12 hrs	1		Ref	
12 hrs and more	1.20(0.42, 3.46)	0.733	0.10(-0.34, 0.55)	0.649
Consecutive days spent at work				
8	1		Ref	

8-14 days	1.43(0.60, 3.41)	0.414	-0.08(-0.46, 0.29)	0.665
15+ days	060(0.13, 2.74)	0.511	-0.19(-0.89, 0.51)	0.589
Consecutive days spent at home				
8 days	1		Ref	
8-14 days	1.29(0.48, 3.47)	0.613	-0.01(-0.44, 0.43)	0.966
Poor sleep condition	1.06(0.42, 2.67)	0.906	0.42(0.06, 0.78)	0.024
Risky alcohol use	1.12(0.47, 2.68)	0.801	0.24(-0.09, 0.57)	0.159
Smoking	1.23(0.48, 3.15)	0.672	0.13(-0.24, 0.49)	0.495
Poor diet	1.92(0.34, 10.92)	0.460	0.39(-0.49, 1.27)	0.385
Weight problems	0.97(0.39, 2.45)	0.954	0.30(-0.09, 0.70)	0.131
Insufficient physical activity	1.50(0.64, 3.48)	0.351	0.32(-0.02, 0.66)	0.062
Poor physical health	5.17(1.18, 22.54)*	0.029	0.82(0.38, 1.26)	<0.001
Psychological distress	4.14(1.55, 11.08)**	0.005	0.50(0.12, 0.87)	0.010
-2 Log likelihood or regression *F* statistic	-126.347		F(26, 89)=1.99	
P value for -2 log likelihood or regression P value	0.010		0.001	
Pseudo R^2/adjusted R^2	0.153		0.184	

Adjusted for age, sex and co-occurrence of multiple health risk

Table S4c. Health and work-related predictors of any total productivity loss (based on logistic regression using the total study sample) and percent total productivity loss (based on least squares regression limited to study sample with positive total productivity loss)

Parameters	Logistic regression of any total productivity loss (N=216)		Least square regression of percent total productivity loss (n=121)	
	OR(95%CI)	p-value	β(95%CI)	p-value
Age in years				
<35	1		Ref	
35-44	0.60(0.26, 1.38)	0.230	-0.03(-0.46, 0.41)	0.909
45-54	0.73(0.25, 2.08)	0.553	-0.14(-0.66, -0.38)	0.602
55+	0.38(0.12, 1.17)	0.092	-0.02(-0.69, 0.65)	0.952
Sex				
Male	1			
Female	1.03(0.48, 2.20)	0.943	-0.03(-0.43, 0.36)	0.879
FIFO role				
Management	1		Ref	
Professional	1.50(0.51, 4.41)	0.458	0.36(-0.22, 0.95)	0.226
Maintenance/Technician	2.00(0.71, 5.58)	0.188	0.30(-0.25, 0.85)	0.287
Production/Drilling/Construction/Labourer	1.75(0.69, 4.42)	0.239	0.24(-0.26, 0.75)	0.348
Machinery operator and driver	2.25(0.76, 6.59)	0.142	0.29(-0.27, 0.85)	0.314
Catering/Other	1.70(0.43, 6.67)	0.445	0.16(-0.59, 0.91)	0.677
FIFO duration in years				
5	1		Ref	
5-9	0.93(0.40, 2.18)	0.872	-0.27(-0.69, 0.16)	0.217
10+	0.48(0.21, 1.12)	0.091	-0.30(-0.75, 0.15)	0.195
Shift pattern				
Regular shift	1		Ref	
Rotation shift/other	1.41(0.70, 2.85)	0.334	0.19(-0.15, 0.53)	0.274
Shift hours				
12 hrs	1		Ref	
12 hrs and more	1.40(0.48, 4.06)	0.535	0.14(-0.41, 0.70)	0.616
Consecutive days spent at work				

8	1		Ref	
8-14 days	1.38(0.58, 3.29)	0.471	-0.22(-0.68, 0.24)	0.357
15+ days	0.65(0.15, 2.89)	0.574	-0.20(-1.05, 0.65)	0.643
Consecutive days spent at home				
8 days	1		Ref	
8-14 days	1.31(0.49, 3.52)	0.589	-0.06(-0.60, 0.48)	0.834
Poor sleep condition	1.06(0.42, 2.69)	0.897	0.41(-0.05, 0.87)	0.080
Risky alcohol use	1.03(0.43, 2.48)	0.945	0.27(-0.14, 0.69)	0.199
Smoking	0.85(0.33, 2.20)	0.737	0.13(-0.32, 0.58)	0.576
Poor diet	2.19(0.40, 12.13)	0.370	0.64(-0.48, 1.75)	0.262
Weight problems	0.90(0.36, 2.26)	0.820	0.24(-0.25, 0.74)	0.337
Insufficient physical activity	1.34(0.57, 3.13)	0.501	0.46(0.04, 0.89)	0.034
Poor physical health	4.02(0.94, 17.20)	0.061	0.87(0.32, 1.43)	0.002
Psychological distress	2.85(1.07, 7.57)	0.035	0.54(0.08, 1.00)	0.021
-2 Log likelihood or regression *F* statistic	-126.048		F(26, 94)=1.62	
P value for -2 log likelihood or regression P value	0.014		0.049	
Pseudo R^2 or adjusted R^2	0.149		0.118	

Adjusted for age, sex and co-occurrence of multiple health risk
